# Cortical Strut Graft for Enigmatic Thigh Pain in Uncemented Total Hip Replacement

**DOI:** 10.7759/cureus.8233

**Published:** 2020-05-22

**Authors:** Luke Granger, Marcus Bankes, Nemandra A Sandiford

**Affiliations:** 1 Orthopaedics, Leicester Orthopaedic Rotation (East Midlands South), Leicester, GBR; 2 Trauma and Orthopaedics, Guy's and St Thomas' Hospital, London, GBR; 3 Joint Reconstruction Unit (Hip and Knee), Southland Teaching Hospital, Invercargill, NZL

**Keywords:** total hip replacement, uncemented, strut graft, cortical, allograft, thigh pain, stem tip

## Abstract

Aims

Enigmatic thigh pain in uncemented femoral components of a total hip replacement can be severe and disabling. Treatment can be conservative or surgical with cortical strut graft or revision of the femoral stem. Cortical strut grafting may offer good results with reduced morbidity. The aim of this study was to report the functional and radiographic outcomes of four patients with enigmatic thigh pain treated with cortical strut allograft.

Materials and Methods

Between 2016 and 2018, four women underwent cortical strut allografting at two centres. All patients had an uncemented, proximally porous S-ROM femoral implant (DePuy, Warsaw, In, USA). All other causes of anterolateral thigh pain were excluded. The mean age was 36.7 years (range: 29-51 years). Patients were followed up for a minimum of 14 months (range: 14-38 months). The University of California, Los Angles (UCLA) activity score, pain scores, complications, and radiographs at six weeks, three months, six months, nine months and one year were recorded.

Results

Mean UCLA activity scores increased from 3.2 (range: 2-4) to 6.2 (range: 6-7) post-operatively. Radiologically, all four patients had complete osseointegration of their strut grafts. Pain scores decreased at six weeks and at six months. One deep venous thrombosis occurred. One patient experienced recurrence of anterolateral thigh pain 26 months post-strut graft, which resolved with protected weight-bearing and analgesia for three months.

Conclusions

In uncemented femoral prostheses, cortical strut grafting to treat enigmatic thigh pain can reduce symptoms and increase activity without the need to revise a well-fixed femoral stem. We add to the growing body of evidence that this can be a successful surgical technique.

## Introduction

Enigmatic thigh pain is a recognised complication following total hip replacement (THR) using uncemented femoral components. The incidence ranges from 0.5% to 40% [1-5]. A smaller percentage of patients experience severe, disabling thigh pain (1.4%-4%) [3].

It is a diagnosis of exclusion and should only be made after other more common pathology is ruled out. These include factors occurring at the bone prosthesis interface (infection, fracture or loosening), soft tissue inflammation (quadriceps strain, iliopsoas tenosynovitis or trochanteric bursitis) and distant sites of referred pain (radiculopathy, spinal stenosis).

Pain is thought to arise secondary to a Young’s modulus mismatch between the host femur and the relatively stiff implant, resulting in a stress riser at the stem tip [3,4,6]. Periosteal irritation, micromotion at the bone-prosthesis interface through an unstable implant and periosteal hypertension are other hypotheses [7].

Surgical and conservative management options have been described [3]. Non-steroidal anti-inflammatory drugs (NSAIDs), protected weight-bearing and activity modification are recommended for one to two years in order for the femur to fully remodel to the new stresses. If pain continues and surgical treatment is required, cortical strut allograft or femoral stem revision may be performed. Strut grafting offers the potential for good functional results with reduced morbidity compared with revision of a well-fixed stem. This is particularly desirable for younger patients as it is a bone-conserving option and mitigates the risks associated with the revision of an existing prosthesis, such as infection and instability. There is a paucity of evidence examining the results of this technique, however.

The aim of this study is to report the functional and radiological outcomes of four patients who underwent cortical strut allografting for recalcitrant thigh pain with well-fixed S-ROM femoral components (DePuy, Warsaw, In, USA).

## Materials and methods

Four female patients underwent cortical strut allograft procedures between 2016 and January 2018. The diagnosis in all cases was unresolved severe thigh pain following THR using the S-ROM femoral implant. These procedures were performed by experienced surgeons across two institutions. Mean age at surgery was 36.7 years (range: 29-51 years). All patients had S-ROM femoral components and a Pinnacle acetabular component (Depuy Synthes) with ceramic on ceramic bearings. The S-ROM implant was used as a primary THR in three cases and as a revision in one. Indications were hip degeneration secondary to slipped capital femoral epiphysis (two patients), developmental dysplasia and conversion from an excision arthroplasty to THR following treatment for prosthetic joint infection. Clinical and radiological reviews were undertaken at 6 weeks, 3 months, 6 months and 12 months post-operatively and annually thereafter. Data collected at the final follow-up were the University of California, Los Angles (UCLA) activity score, pain scores and complications. All patients consented for their information to be used anonymously in research.

Patient selection

**Figure 1:**
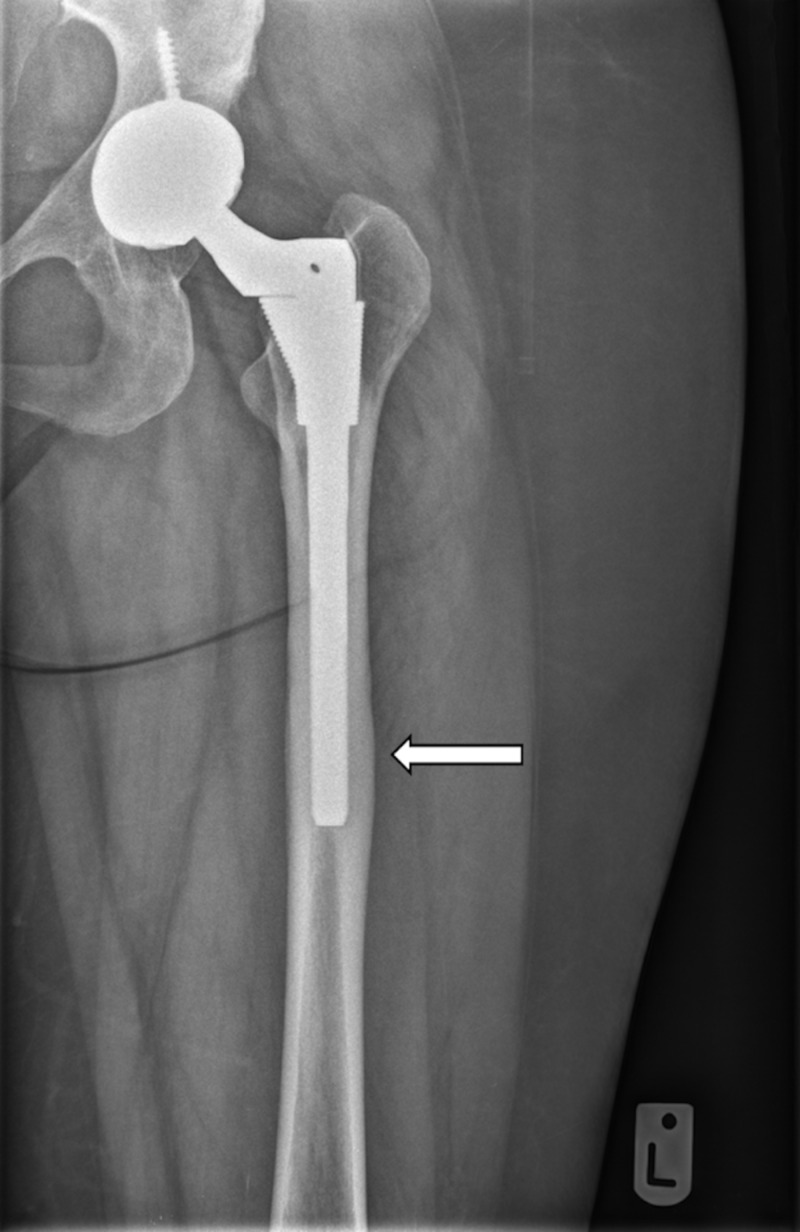
AP radiograph of left hip, S-ROM femoral stem and Pinnacle acetabular cup, with cortical hypertrophy in zone 3 (white arrow). AP, anteroposterior

**Figure 2:**
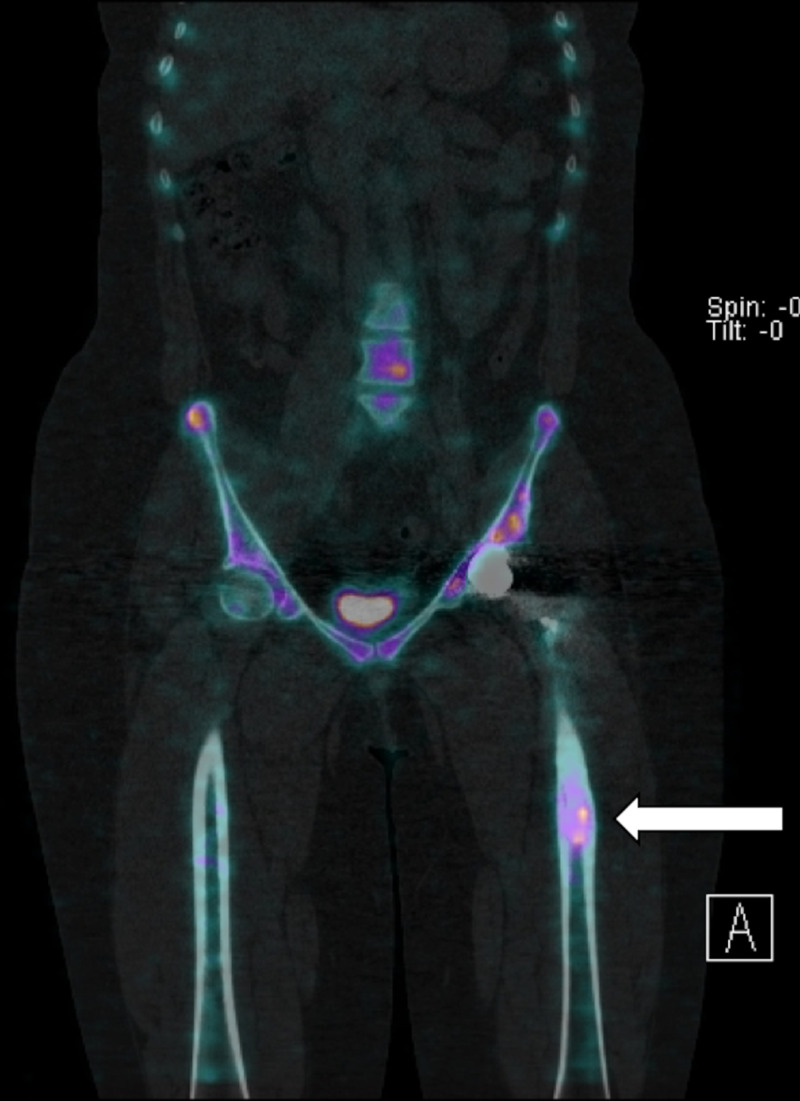
SPECT scan in the coronal view of the trunk and lower limbs. High-intensity uptake is seen in the stem-tip region of the left femur (white arrow). SPECT, single-photon emission computed tomography

All four patients reported thigh pain at six months following their hip arthroplasty procedure. All patients had experienced an uneventful post-operative recovery and had experienced satisfactory progress in rehabilitation prior to this. Their pain was gradual in onset, severe and affected their activities of daily living. The pain was localised to the tip of the femoral component in all cases. Other causes were excluded through history, examination and investigations. On examination, all patients had full range of movement in their hip, and the hip joint was not irritable. Pre-operative investigations included plain radiographs, inflammatory markers (white cell count [WCC], C-reactive protein [CRP] and erythrocyte sedimentation rate [ESR]) and single-photon emission computed tomography (SPECT) scan. In all cases the components were well fixed, and WCC, CRP and ESR were normal. All radiographs showed cortical remodelling in zone 3 (Figure 1) [8]. The SPECT scans showed no loosening of the femoral component and high signal uptake in the stem-tip region corresponding to the focus of pain (Figure 2). All SPECT scans were performed at least one year post-operatively. Initially, the patients were treated with analgesia, activity modification and protected weight-bearing for 12 months. Cortical strut grafting was proposed when the thigh pain did not resolve with conservative measures and continued to affect their activities of daily living.

Operative technique

Fresh frozen diaphyseal femoral allograft was used in all four cases. All procedures were performed by orthopaedic surgeons experienced in arthroplasty (N. A. S., M. J. K. B.). The skin incision was made using an image intensifier to locate the stem tip. A sub-vastus approach to the femur was used. Four cerclage cables were passed: two above the stem tip and two below. The periosteum was stripped to expose the cortex, and a 15-cm fresh frozen diaphyseal femoral strut graft was contoured to fit the patient’s femur using a high-speed burr. The inner surface and edges of the strut graft were coated with demineralised bone matrix (DBX) and placed on the lateral side of the femur, centred on the stem tip. Morsellised bone graft was placed in the interface between the allograft and the native femur. The position of the strut graft was confirmed on an image intensifier and was then secured with the cables. All patients received three doses of intravenous antibiotic prophylaxis (cefuroxime) and subcutaneous low molecular weight heparin (LMHW) until discharge. All patients were allowed to weight-bear as tolerated with crutches following surgery.

## Results

**Figure 3:**
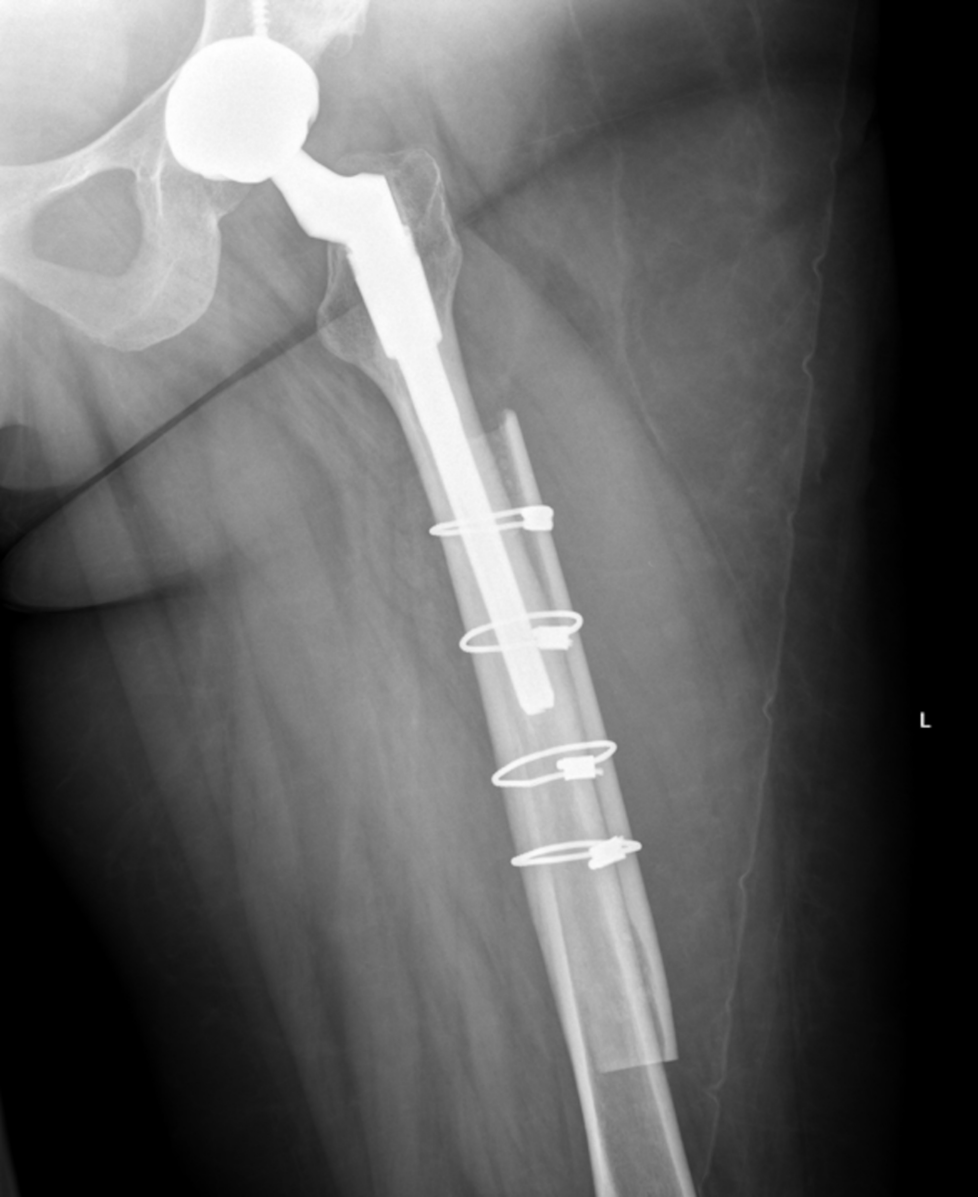
AP radiograph one day post-operatively of the left femur cortical strut allograft. AP, anteroposterior

**Figure 4:**
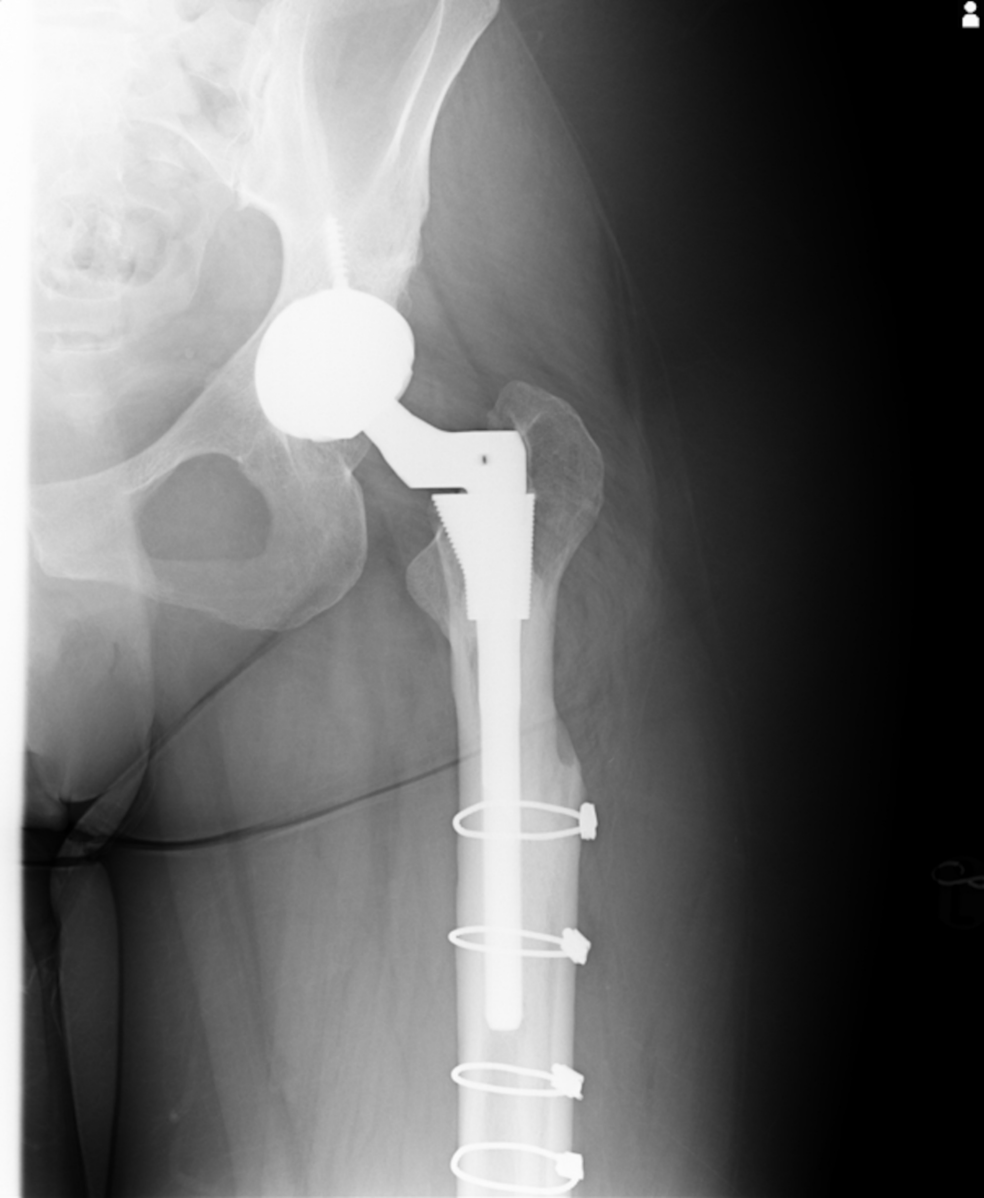
AP radiograph one year post-operatively of the left femur cortical strut allograft. AP, anteroposterior

Minimum follow-up was 14 months (range: 14-38 months). All patients reported improvement in thigh pain at six weeks and almost complete resolution by six months post-operatively. The mean pre-operative UCLA activity score at the final follow-up increased from 3.2 (range: 2-4) to 6.2 (range: 6-7) post-operatively. Radiologically, all four patients had complete osseointegration of their strut grafts (Figures 3, 4). There were no cases of cerclage wire breakage or fracture of the allograft.

There were no complications intra-operatively and no cases of wound breakdown, deep infection, peri-prosthetic fracture or neurovascular injury. One patient developed a deep vein thrombosis (DVT) post-operatively and was treated successfully with rivoraxaban 20 mg once a day for six months. There were no re-operations.

One patient experienced recurrence of thigh pain 26 months post-strut grafting. There was no history of trauma, and the pain was located in the mid-thigh. The pain was brought on following long walks or moderate activities and relieved by rest. At this point, she had returned to her normal life and level of activity.

On examination, the hip had full range of movement and was not irritable. There was no point tenderness at the stem tip. The operative scar was healthy, inflammatory markers were normal and the radiographs showed a well-integrated strut graft and well-fixed components. The patient was mobilised partially weight-bearing with crutches for four weeks. The patient reported resolution of pain at her three months later. There has been no recurrence of pain.

## Discussion

To our knowledge, this is the first report of cortical strut grafting for recalcitrant thigh pain in patients with well-fixed S-ROM femoral stems. All patients had a satisfactory resolution of pain and returned to full function following surgery. Management of unresolved functional thigh pain following total hip arthroplasty can be challenging. Thigh pain after THR often resolves spontaneously. Most patients respond to activity modification, non-steroidal anti-inflammatory drugs and short-term protected weight-bearing. The patients who experience ongoing debilitating thigh pain despite conservative measures might benefit from surgical intervention. The optimal duration for conservative measures is undecided.

Young’s modulus of elasticity in the native femur is approximately 10 times less than titanium alloys (Ti-6Al-4V) and 18 times less than cobalt-chromium (Co-Cr). This results in a modulus mismatch or a significant difference in the structural rigidity at the interface between the tip of the stem and the adjacent bone. It has been postulated that stress transfer to the femur at this point results in enigmatic thigh pain [3].

Other hypotheses suggest the pain arises from periosteal and/or endosteal irritation, with basic science studies demonstrating pain mediators substance P and calcitonin gene related peptide in the bone and soft tissues near the stem tip [7].

Implant characteristics such as diameter and material influence stiffness and have been correlated with increased thigh pain. Vresilovic et al. report 0-15% incidence in the smallest sizes rising to 23-40% in the largest, with 12.1% overall incidence of thigh pain at one year [9]. Burkart et al. found thigh pain with a cobalt-chromium alloy (porous coated anatomic stem) to be 23% at two years versus 3% with a less stiff titanium implant (Mallory-Head® stem, Biomet, Warsaw, IN, USA) [4]. Short-stem THRs (Tri-Lock Bone Preservation Stem, DePuy Synthes) may have even higher rates of thigh pain, up to 25% [10]. Modern uncemented implants are reported to have lower incidences of thigh pain. The Corail® stem (DePuy Synthes), when used in good bone stock, has been described as having as low as 0.6% (2/347) rate of unexplained thigh pain; however, in neck of femur surgery with osteoporotic bone, the rate may be as high as 31.9% [11,12].

The S-ROM prosthesis is a modular, cementless femoral stem consisting of a porous coated proximal titanium sleeve with a conical and spout. The titanium stem is polished distally with integrated splines for rotational stability and a coronal slot to theoretically reduce stem stiffness and the incidence of thigh pain. Thigh pain with this implant is reported between 1.6% and 11.9% [13-16].

Addition of a cortical strut allograft adds rigidity to the femur, which may reduce the difference in structural rigidity between the native femur and the implant and, in turn, reduce the stresses transferred to the femur and the resulting pain caused by this. In addition, denervation of the lateral femoral periosteum when applying the strut graft may also provide pain relief [3].

Femoral strut grafts have been shown to integrate well when used to augment the host femur when revising a THR to an uncemented prosthesis [17]. Their use in the management of enigmatic thigh pain following primary uncemented THR has been described only once previously to our knowledge. Domb et al. presented their findings using this technique in seven patients [18]. Resolution of pain occurred in six cases. One patient who achieved pain relief underwent revision of the femoral stem two years after strut grafting due to aseptic loosening. While all patients showed evidence of some graft uptake, only four of seven patients had full graft incorporation proximally and distally.

We observed pain relief in all cases at the time of the final follow-up. Full incorporation of the graft was also observed in all cases in our cohort. Domb et al. sought to exclude stress fractures at the stem tip using bone scans, which were negative in all patients prior to surgery [18]. SPECT imaging revealed increased uptake at the stem tip without evidence of loosening or fracture. This imaging modality may be more sensitive to increased stress at the stem tip and should therefore be considered during the workup of a patient with enigmatic thigh pain.

One case of DVT occurred post-operatively in our study. The patient was 31 years old, had a body mass index of 35 and took oral contraceptive pills. This was successfully treated with oral anticoagulants. There were no wound healing issues. This illustrates the procedure is not without risk.

One patient in our cohort who achieved pain relief experienced recurrence of thigh pain approximately two years post-strut grafting. This differs from the experience of Domb et al. [18]. The femoral strut graft was well incorporated. The aetiology of this pain is unclear. It was potentially due to the surrounding soft tissues as was successfully managed with restricted weight-bearing and analgesia. In our experience, recurrence after surgery may still be managed conservatively as for a first presentation. This management strategy avoids revision arthroplasty and its associated morbidity in young patients.

The results of this study are limited by small patient numbers and 14 months of minimum follow-up. However, we describe a rare technique with positive short-term outcomes and add to the existing body of evidence that cortical strut grafting can be a successful procedure for enigmatic thigh pain.

## Conclusions

Enigmatic thigh pain in uncemented THRs may be secondary to a Young’s modulus mismatch between the stiff implant and the relatively less stiff femur. Partial weight-bearing with crutches, activity modification and NSAID therapy should be employed for one to two years to allow the femur to remodel to these new stresses. However, should this fail, the results of this study suggest that cortical strut allograft is a viable option for the management of enigmatic thigh pain following uncemented THR. The morbidity associated with this procedure is less than the alternative of revising a well-fixed implant and the associated bone loss.
